# Reliability of Medical Journals from Pakistan: Challenges and way Forward

**DOI:** 10.12669/pjms.40.8.10617

**Published:** 2024-09

**Authors:** Shaukat Ali Jawaid, Jamshed Akhtar, Masood Jawaid

**Affiliations:** 1 Shaukat Ali Jawaid Chief Editor, Pakistan Journal of Medical Sciences Karachi. Pakistan; 2 Prof. Jamshed Akhtar Visiting Faculty National Institute of Child Health, Karachi, Pakistan; 3 Dr. Masood Jawaid Associate Editor, Pakistan Journal of Medical Sciences Karachi. Pakistan

Members of the medical profession, particularly those in teaching institutions, are bound to conduct and publish scientific research papers to meet one of academic requirements for the promotion. This has given birth to “paper mills”, a dark side of academic publishing, encouraged gift authorship as well as host of scientific and ethical misconducts reported from all over the world, Pakistan being no exception. Sale and purchase of authorship has now become a very common phenomenon. This has added to the existing burden of responsibilities on the editors of biomedical journals to check this menace and halt the intellectual corruption. Editors, therefore, have to be competent, and acquire the core competencies.^1^ They must comply with the International Committee of Medical Journals (ICMJE) current authorship guidelines, and COPE guidelines on publication ethics.^2^,^3^ It is mandatory for them to publish policies regarding the editorial contents, peer review process, managing scientific misconduct and others on the journal website to ensure transparency. The websites of ICMJE as well as COPE provide useful information for the training of editors. COPE website has many useful Flow Charts to help manage scientific misconduct.^2^.^3^

Health research is a part of undergraduate medical curriculum in Pakistan. However, the format of teaching and training is not uniform. It is observed that the majority of the healthcare professionals are not aware of the publication process. They only come to realize this when submission of the manuscript is made to the standard peer reviewed journals. Many of the submissions are rejected at initial triage – the desk rejection, as authors fail to understand the scope of the journal or are unable to follow the instructions to the authors. At times the authors have to redress the manuscripts to comply with the format of the journals and more commonly revise the article, maybe multiple times after the peer review, before being accepted for publication. During this process that takes time many authors get frustrated and some of them get trapped by the research paper mills and predatory journals for provision of ready-made manuscripts and quick publication. The submissions of fake research papers to the sub-standard journals, predatory journals and even in indexed journals, whether knowingly or unknowingly, fall into unethical practices. The number of research papers retracted during the year 2023 published in various journals were more than ten thousand which shows how prevalent such a trend is all over the world.^4^

The publication processes of number of medical journals from Pakistan is also not transparent. Recently one of the medical journals from Pakistan has been removed from international databases Web of Science by Clarivate because of dubious processes. Unfortunately, the articles published in that journal cannot be accepted for promotion and authors shall get no credit for their hard work. The editors of some of the medical journals published from Pakistan are not fully trained in the art of scientific writing, peer review requirement and publication. A lack of professionalism on the part of the editors is apparent as they keep on entertaining and publishing sub-standard research work that amounts to quackery in medical journalism. Sadly, they also get encouragement when these journals also get recognition from the regulatory bodies of Pakistan because of lack of oversight or for some other reasons. It is pertinent to mention here that two regulatory bodies for medical journals tried to streamline the process of recognition through journal evaluation committees. However, some members of these committees are either not professional editors or well versed with the process of publication that involves multiple coordinated efforts, done both scientifically as well ethically in a rigorous manner. The editors of journals with dubious publication processes must be aware of the facts that published articles are in the public domain. The flawed data and write up of these manuscripts can be easily picked up by any person with a minimum scientific knowledge. The integrity of the members of the scientific committee therefore is also at stake. It is important that minutes of the journal evaluation committees must be made public through the website against each journal. The observations made by the members are intended to make the publication process a transparent endeavor and to improve its quality. This will facilitate researchers to choose appropriate journals for the submission of their manuscript. It is time to re-visit all the previously recognized journals as unethical practices are reported frequently. By publishing in sub-standard journals approved by the regulatory authorities, a large number of faculty members got promoted. This is going to have serious implications for teaching, training as well as patient care.

Pakistan Association of Medical Editors (PAME) 5 which is a registered body of medical editors in Pakistan had realized the importance of training the editors many years ago. Hence in collaboration with University of Health Sciences (UHS)^6^ Lahore, it started a Certificate Course in Medical Editing (CME) and an Advance Course in Medial Journalism and Editing (CMJE) leading to Diploma in Medical Journalism in 2019. These courses are of six-month duration with two contact sessions of four days each. During the course the participants are taught by distinguished medical editors and the content covers a vast area, from initial screening or Editor’s triage to copy editing, peer review processes, identifying and handling scientific misconduct, indexing in various databases, Scientometrics, basic principles of scientific writing, different business models, generation of funds to sustain the journals and others. Use of online journal management system (OJS) is also taught and hands on exercises are given throughout in-person sessions. Each student is mentored by an eminent researcher and editor.

The assessment process is based upon the principles of adult learning. Students are given assignments that include reflective writing, writing for a journal, be it an editorial or any other category of manuscript, and submission of a portfolio that is evaluated by independent academicians and researchers. They are also evaluated by their mentors. The final assessment is done through viva examination by an internal and a pair of external examiners. Those who earn at least 60% of the marks are declared successful and get a certificate. To date 185 editors, editorial staff members, editorial board members and faculty members from all over the country have qualified and earned this certificate. The details appear in Table-I.

**Table-I:** Participants in Certificate Course in Medical Editing (Batch-1 to Batch-6).

Male	89
Female	96
Punjab	163
Sindh	9
KPK	10
Baluchistan	3

Total:	185

Many of them are now affiliated with various medical journals and have helped improve their quality and standard to a great extent. This is one of the accomplishments of which PAME is proud of.

The advance course also includes facilitators, Prof. Noshina Saleem and Prof. Mian Abdul Hanan Ahmed from the School of Communication Studies of University of Punjab. They discuss in detail different aspects of Health Journalism in print and electronic media to give a broader view. A biostatistician is also involved to teach basics of data entry and statistics involved in the research and publication. The teaching material is provided to the candidates for both the courses. Constant efforts are being made to further improve the content. More recently the subject of the use of Artificial Intelligence in scientific writing was included.

The second contact session in advance course consists of four days internship at Impact Factor Journals in Karachi i.e. Pakistan Journal of Medical Sciences, Journal of Pakistan Medical Association and Journal of College of Physicians & Surgeons Pakistan. This provides them an opportunity to observe and have firsthand information about the working of these three journals. They are required to interact and understand the different peer review systems and business models of these journals and the same can be applied to the journals they work for. So far two batches have completed this course. There were twelve candidates in the first batch and seven in the second batch, which has just completed their training in June this year. Now after viva examination and portfolio evaluation they are supposed to write a research report to become eligible for Diploma in Medical Journalism.

It is to be noted that there are other candidates who due to various reasons cannot join these Face-to-Face training courses. Hence to facilitate them, PAME and Pakistan Journal of Medical Sciences, KEMU are collaborating with CORTEACH in running this CMEJ Online Course. At present the 5^th^ batch is enrolled and is being trained.^7^ This online course has a structured format of teaching with assignments and continuous evaluation. So far 189 healthcare professionals have attended the online course. Table-II.

**Table-II:** Participants in CORTEACH Online Course.

Male	104
Female	85
Punjab	89
Sindh	42
KPK	52
Baluchistan	1
Saudi Arabia	3
Bahrain	2

Total:	189

Face to Face learning offers many advantages including constant feedback and monitoring that is considered ideal. Online learning is also very convenient for many healthcare professionals. It is extremely important that candidates of both the format of these courses also practice what is taught to them during the sessions.

PAME, with its meagre resources, has tried to provide these training opportunities to newly appointed editors, editorial staff members of journals, researchers and faculty members interested to improve their professional capabilities. If some editors in their wisdom do not attend or practice what they have learnt, one cannot help them. Having produced this human resource, now PAME intends to ask the regulatory bodies to make sure that the editors of all the biomedical journals must attend these courses or similar programs to address the menace of quackery in medical journalism.

There are reports of PM&DC and HEC planning to form a joint committee for the assessment, evaluation and recognition of the medical journals. This is a welcome step and deserves appreciation. PAME is also planning to start evaluation and recognition of biomedical journals for which a committee of distinguished senior editors will be formed. It is hoped this committee will work to keep up professional ethics upfront and not influenced by external pressures. All these efforts by PAME are directed to ensure improvement of the quality of manuscripts accepted for publication and standard of the biomedical journals published from Pakistan which will also improve Pakistan’s contribution to world literature on healthcare.
